# Development of Gateway Binary Vector Series with Four Different Selection Markers for the Liverwort *Marchantia polymorpha*

**DOI:** 10.1371/journal.pone.0138876

**Published:** 2015-09-25

**Authors:** Kimitsune Ishizaki, Ryuichi Nishihama, Minoru Ueda, Keisuke Inoue, Sakiko Ishida, Yoshiki Nishimura, Toshiharu Shikanai, Takayuki Kohchi

**Affiliations:** 1 Graduate School of Biostudies, Kyoto University, Kyoto, Japan; 2 Graduate School of Science, Kobe University, Kobe, Japan; 3 Department of Botany, Graduate School of Science, Kyoto University, Kyoto, Japan; University of Tsukuba, JAPAN

## Abstract

We previously reported *Agrobacterium*-mediated transformation methods for the liverwort *Marchantia polymorpha* using the hygromycin phosphotransferase gene as a marker for selection with hygromycin. In this study, we developed three additional markers for *M*. *polymorpha* transformation: the gentamicin 3'-acetyltransferase gene for selection with gentamicin; a mutated acetolactate synthase gene for selection with chlorsulfuron; and the neomycin phosphotransferase II gene for selection with G418. Based on these four marker genes, we have constructed a series of Gateway binary vectors designed for transgenic experiments on *M*. *polymorpha*. The 35S promoter from cauliflower mosaic virus and endogenous promoters for constitutive and heat-inducible expression were used to create these vectors. The reporters and tags used were Citrine, 3×Citrine, Citrine-NLS, TagRFP, tdTomato, tdTomato-NLS, GR, SRDX, SRDX-GR, GUS, ELuc(PEST), and 3×FLAG. These vectors, designated as the pMpGWB series, will facilitate molecular genetic analyses of the emerging model plant *M*. *polymorpha*.

## Introduction

Bryophytes, including liverworts, mosses, and hornworts, are the earliest diverging group of extant land plants. Liverworts are a key lineage for comparative genomic studies aimed at elucidating the fundamental and diverse gene regulatory networks in land plants [1–3]. The liverwort *Marchantia polymorpha* L. is a widespread dioecious species that is easily cultivated in laboratory conditions. Previous research on *Marchantia* has focused on the genomes of organelles in cultured cells [4,5] and its Y chromosome [6]. Recently, the whole genome shotgun sequencing project of *M*. *polymorpha* was initiated under the Community Sequencing Program at the Joint Genome Institute (DOE-JGI: http://jgi.doe.gov/sequencing/why/CSP2008/mpolymorpha.html). Our research group has been developing *M*. *polymorpha* as an experimental model organism, because it occupies a critical evolutionary position for studies on specific molecular and cellular developmental processes in detail [7].

We previously reported an efficient *Agrobacterium*-mediated transformation system using sporelings of the liverwort *M*. *polymorpha*. Hundreds of transformants carrying T-DNAs randomly inserted in the genome were obtained from a single sporangium [8]. An *Agrobacterium*-mediated transformation method has also become available using thallus explants in *M*. *polymorpha*, providing opportunities for genetic transformation without preparing spores [9]. Recently, these methods have been simplified so that all of the procedures can be conducted in a Petri dish [10,11]. These methods for genetic transformation have been used for functional molecular studies of *M*. *polymorpha* [12–24]. Furthermore, a method for homologous recombination-mediated gene targeting has been established for *M*. *polymorpha*—this method uses positive/negative selection in combination with an efficient *Agrobacterium*-mediated transformation system [25]. To date, however, only the hygromycin phosphotransferase gene (*hpt*) has been available for use as a marker for *Agrobacterium*-mediated transformation in *M*. *polymorpha*. Introduction of a transgene into pre-existing transgenic lines is an important method for analyses of transgene function and the localization of its product. For this purpose, additional selection markers that differ from those in pre-existing transgenic plants are required.

Here we describe three markers that have been developed for *M*. *polymorpha* transformation: the gentamicin 3'-acetyltransferase gene (*aacC1*) [26] for selection with gentamicin; a mutated acetolactate synthase gene (*mALS*) for selection with the sulfonylurea herbicide, 2-chloro-N-[(4-methoxy-6-methyl-1,3,5-triazin-2-yl)aminocarbonyl]-benzenesulfonamide (chlorsulfuron; CS); and the neomycin phosphotransferase II (*nptII*) gene for selection with G418. Based on these marker genes, we have designed and constructed a series of Gateway binary vectors, the pMpGWB series, for transgenic experiments on *M*. *polymorpha*. Using a combination of different selection markers, multiple constructs can be introduced into *M*. *polymorpha* by a single transformation procedure. These pMpGWB vectors will facilitate transgenic research on the model plant *M*. *polymorpha*.

## Materials and Methods

### Plant material and growth conditions

Male and female accessions of *M*. *polymorpha*, Takaragaike-1 and Takaragaike-2, respectively, were asexually maintained as previously described [8]. Plants were cultured on half-strength Gamborg’s B5 medium [27] containing 0.5 g/L MES and 1% (w/v) agar under continuous white fluorescent light (50–60 μmol photons m^-2^ s^-1^) at 22°C. For transformation, we used F1 spores generated by crossing Takaragaike-1 and Takaragaike-2, prepared as previously described [28].

### Vector construction

All the primers used in this study are listed in S1 Table. The PCR-amplified regions and ligation junctions were confirmed by sequence analysis for all vectors.

Acetolactate synthase (ALS) is a key enzyme in the biosynthesis of the branched-chain amino acids leucine, isoleucine, and valine. Sulfonylurea herbicides, *e*.*g*. CS, bind reversibly to the ALS-FAD-thiamine pyrophosphate-Mg^2+^-decarboxylated pyruvate complex and also compete for the second pyruvate binding site in ALS [29]. Mutagenesis at these sites in ALS confers tolerance to sulfonylurea herbicides. For example, *mALSs* containing mutations at P197H, R198S, and W574L conferred resistance to CS in Arabidopsis [30]. To generate a CS-resistance marker gene for use in *M*. *polymorpha*, its *ALS* sequence was mutagenized to contain corresponding mutations (P207S/R208S/W582L) to those described above, as follows. First, *M*. *polymorpha ALS* cDNA was amplified by RT-PCR with the primer set ALS-P1 and ALS-P2 using first-strand cDNA synthesized from a 10-day-old thallus. The resultant *ALS* cDNA was cloned into the pENTR/D-TOPO vector (Life Technologies, Gaithersburg, MD, USA). A point mutation at W582L in the *ALS* sequence was introduced by PCR-based site-directed mutagenesis with the primer pair ALS-W582L-F and ALS-W582L-R, using the *ALS* cDNA plasmid as the template. The PCR product was digested with *Dpn*I, and the digested PCR product was transformed into *Escherichia coli* competent cells (DH5α). The mutation in *ALS* was confirmed by sequencing the resultant plasmid. The mALS was prepared by repeating the same procedure with the primer set ALS-P207S-R208S-F and ALS-P207S-R208S-F using the W582L-mutated *ALS* as a template. The *mALS* cDNA was cloned into pCAMBIA1300 to replace the *hpt* gene, generating the plasmid pCAMBIA1300-mALS.

The marker cassettes _*pro*_ *35S×2*:*hpt*:_*ter*_ *35S*, _*pro*_ *35S×2*:*aacC1*:_*ter*_ *35S*, _*pro*_ *35S×2*:*mALS*:_*ter*_ *35S*, and _*pro*_ *35S×2*:*nptII*:_*ter*_ *35S* were prepared by PCR with the primer set Marker_LinF and Marker_RinF using pCAMBIA1300, pPZP221, pCAMBIA1300-mALS, and pCAMBIA2301, respectively, as the template. The marker cassettes were cloned into *Eco*RI-digested pGWB400 [31] using an In-Fusion HD cloning kit (Clontech, Mountain View, CA, USA) to replace the _*pro*_ *NOS*:*nptII*:_*ter*_ *NOS* cassette, generating pMpGWB100, pMpGWB200, pMpGWB300, and pMpGWB400, respectively. The *E*. *coli* DH5α cells harboring these plasmids were selected on LB medium containing 100 mg/l spectinomycin.

The Gateway-compatible binary vectors pMpGWBs were constructed using the same strategy as that used by Nakagawa et al. to construct pGWBs [31,32]. Detailed procedures of pMpGWBs construction are described in S1 Text. The *E*. *coli* (strain DB3.1) cells harboring pMpGWBs were selected on LB media containing 100 mg/l spectinomycin and 30 mg/l chloramphenicol.

The vector for expression of TagRFP-LTI6b was constructed as follows: the GFP-LTI6b coding sequence was PCR-amplified using the primers GFP-LTI6b_GW_L1 and GFP-LTI6b_GW_R1 and cloned into pENTR/D-TOPO. The GFP coding sequence flanked by two *Not*I sites in this plasmid was replaced with the TagRFP-coding sequence with two similarly flanking *Not*I sites, which was PCR-amplified using the primers pENTRD_NotI_TagRFP_F and TagRFP_NotI_R. This plasmid was used for LR recombination with pMpGWB103 to generate pMpGWB103-TagRFP-LTI6b.

To construct the vector for expression of SP-GFP-HDEL, the SP-GFP-HDEL-coding sequence [33] was PCR-amplified using the primers SP_Lc and HDEL_R and cloned into pENTR/D-TOPO. The resulting vector was used for LR recombination with pMpGWB303 to generate pMpGWB303-SP-GFP-HDEL.

To construct the vectors for expression of tdTomato-NLS and GUS under the endogenous *ELONGATION FACTOR1α* promoter (_pro_EF), the 1,729-bp promoter sequence of Mp*EF1α* [34] was amplified by PCR using the primers MpEF-P_L1 and MpEF-P_R1 and cloned into pENTR/D-TOPO. The resulting vector was used for LR recombination with pMpGWB216 and pMpGWB404 to generate pMpGWB216-proEF and pMpGWB404-proEF, respectively.

### *Agrobacterium*-mediated transformation of *M*. *polymorpha*

Transformation of *M*. *polymorpha* was performed as described previously [8]. For simultaneous transformation with multiple vectors, the respective *Agrobacterium* cultures were mixed at a 1:1 ratio. Half-strength Gamborg’s B5 medium containing 0.5 g/L MES and 1% (w/v) agar and supplemented with hygromycin B (Wako Pure Chemical Industries, Osaka, Japan), gentamicin sulfate (Nacalai Tesque, Kyoto, Japan), CS (kindly provided by Dupont, Wilmington, DE, USA), or G418 (Nacalai Tesque) was used to select transformants. The stock solution of CS (50 mM) was prepared with DMSO.

### Fluorescence microscopy

TagRFP, GFP, and chlorophyll were excited with a laser at 488 nm and 543 nm, and fluorescence was detected at 500–530 nm (GFP), 580–600 nm (TagRFP), and 655–755 nm (chlorophyll) using a confocal microscope (FV1000; Olympus, Tokyo, Japan).

### GUS staining

Histochemical staining of GUS was performed as described previously [13,35].

### Nucleotide accession numbers and distribution of pMpGWBs

The complete nucleotide sequences of the pMpGWBs have been deposited in the GenBank/EMBL/DDBJ databases under the accessions nos. LC057442 to LC057589. pMpGWB plasmids can be obtained from Addgene (www.addgene.org; plasmid numbers 68062 and 68555–68701).

## Results and Discussion

### Selection of transformants using gentamicin, CS, and G418

To explore the possibility of using gentamicin, CS, and G418 to select transformed *M*. *polymorpha*, we first investigated whether these substances suppressed plant growth. *M*. *polymorpha* gemmae were grown on agar medium containing various concentrations of gentamicin, CS, and G418. Thalli showed severe growth defects when grown on medium containing 50 mg/l or higher concentrations of gentamicin, 0.05 μM or higher concentrations of CS, and 2.5 mg/l or higher concentrations of G418 (S1 Fig).

To facilitate the selection of transformants on medium containing these antibiotics/herbicide, chimeric marker gene cassettes were introduced into the binary vector pGWB400 [31]. The backbone of this vector was pPZP221, harboring ColE1 and pVS1 plasmid origins for replication in *E*. *coli* and *Agrobacterium*, respectively [36]. In the case of hygromycin selection, the nopaline synthase gene promoter did not drive expression of the *hpt* marker gene as strongly as did the CaMV 35S promoter in *M*. *polymorpha*. Consequently, the nopaline synthase gene promoter resulted in a lower transformation efficiency. To ensure efficient expression of the resistance genes in *M*. *polymorpha*, a double-enhancer version of the CaMV 35S promoter (_*pro*_ *35S×2*) in the pCAMBIA vector (http://www.cambia.org/daisy/cambia/585), which was derived from the pPZP vector [36,37], was used to drive the expression of the marker genes. Thus, the constructed binary vectors were pMpGWB100, pMpGWB200, pMpGWB300, and pMpGWB400, carrying _*pro*_ *35S×2*-driven *hpt*, *aacC1*, *mALS*, and *nptII*, respectively (S2 Fig).

**Fig 1:**
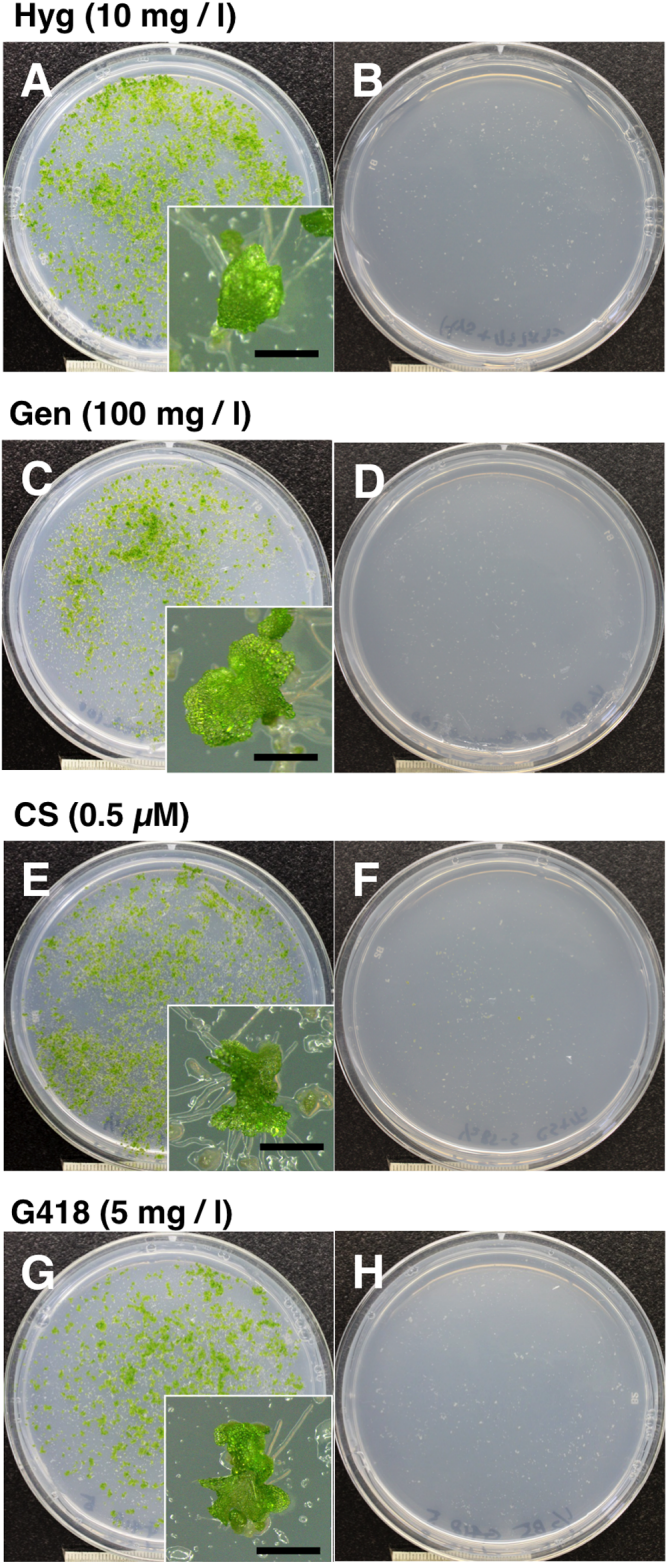
Transformation of *M*. *polymorpha* using pMpGWB vectors developed in this study. Sporelings co-cultivated with *A*. *tumefaciens* strain GV2260 containing the binary vector pMpGWB100 (A), pMpGWB200 (C), pMpGWB300 (E), pMpGWB400 (G), or no binary vector (B, D, F, and H) at 7 d after transfer onto selection medium containing 10 mg/l hygromycin (Hyg; A, B), 100 mg/l gentamicin (Gen; C, D), 0.5 μM chlorsulfuron (CS; E, F), or 5 mg/l G418 (G, H). Insets show magnified view of antibiotic/herbicide-resistant transformants. Bars = 1 mm.

**Fig 2:**
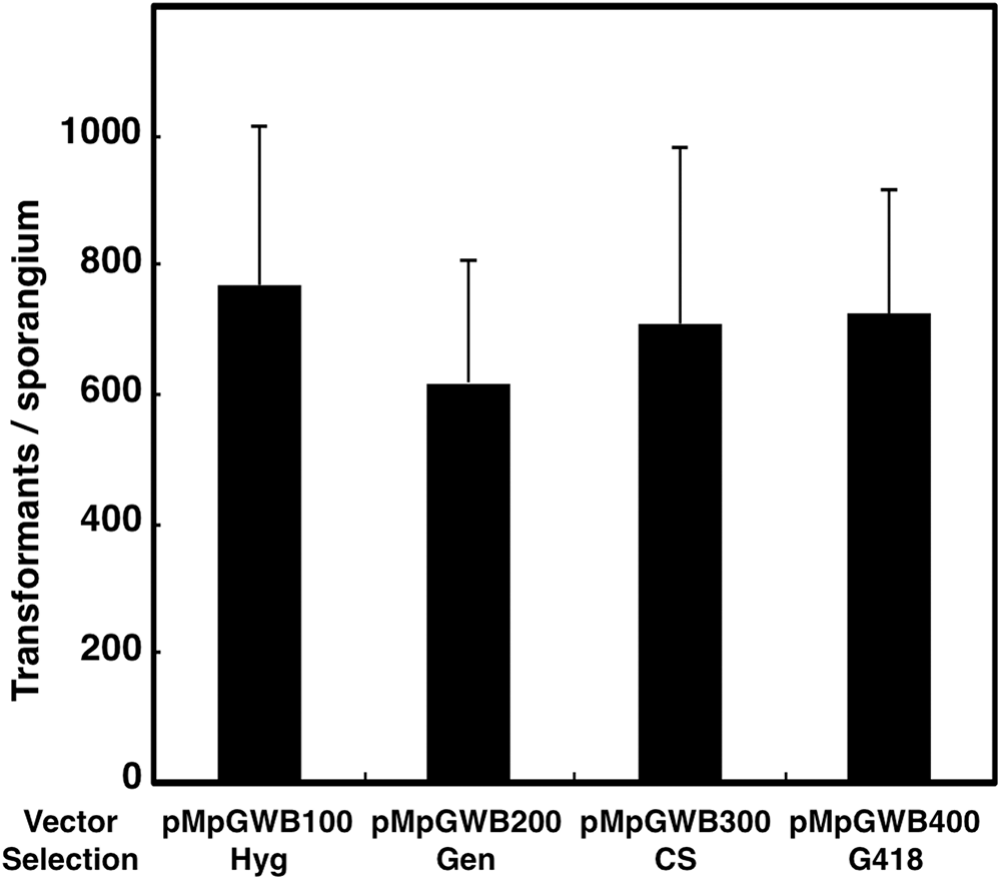
Comparison of transformation efficiencies of *M*. *polymorpha* among four vectors with different selection markers and selective antibiotics/herbicide. Hyg, hygromycin; Gen, gentamicin; CS, chlorsulfuron. Values are means ± SD from three independent experiments.

The vectors pMpGWB100, pMpGWB200, pMpGWB300, and pMpGWB400 were used to transform *M*. *polymorpha* using *Agrobacterium*. Sporelings co-cultured with *Agrobacterium* harboring the respective plasmids gave rise to antibiotic/herbicide-resistant plantlets on medium containing 10 mg/l hygromycin, 100 mg/l gentamicin, 0.5 μM CS, or 5 mg/l G418, respectively (Fig 1A, 1C, 1E and 1G), whereas the sporelings co-cultured with *Agrobacterium* carrying no binary plasmid did not (Fig 1B, 1D, 1F and 1H). The transformation efficiencies obtained via gentamicin/_*pro*_ *35S×2*::*aacC1*::_*ter*_ *35S*, CS/_*pro*_ *35S×2*::*mALS*::_*ter*_ *35S*, and G418/_*pro*_ *35S×2*::*nptII*::_*ter*_ *35S* selection were comparable to that obtained by hygromycin/_*pro*_ *35S×2*::*hpt*::_*ter*_ *35S* selection (Fig 2). The antibiotic/herbicide-resistance traits were transmitted through asexual reproduction of gemmae in multiple rounds of subculture.

### Gateway binary vectors pMpGWBs

**Fig 3:**
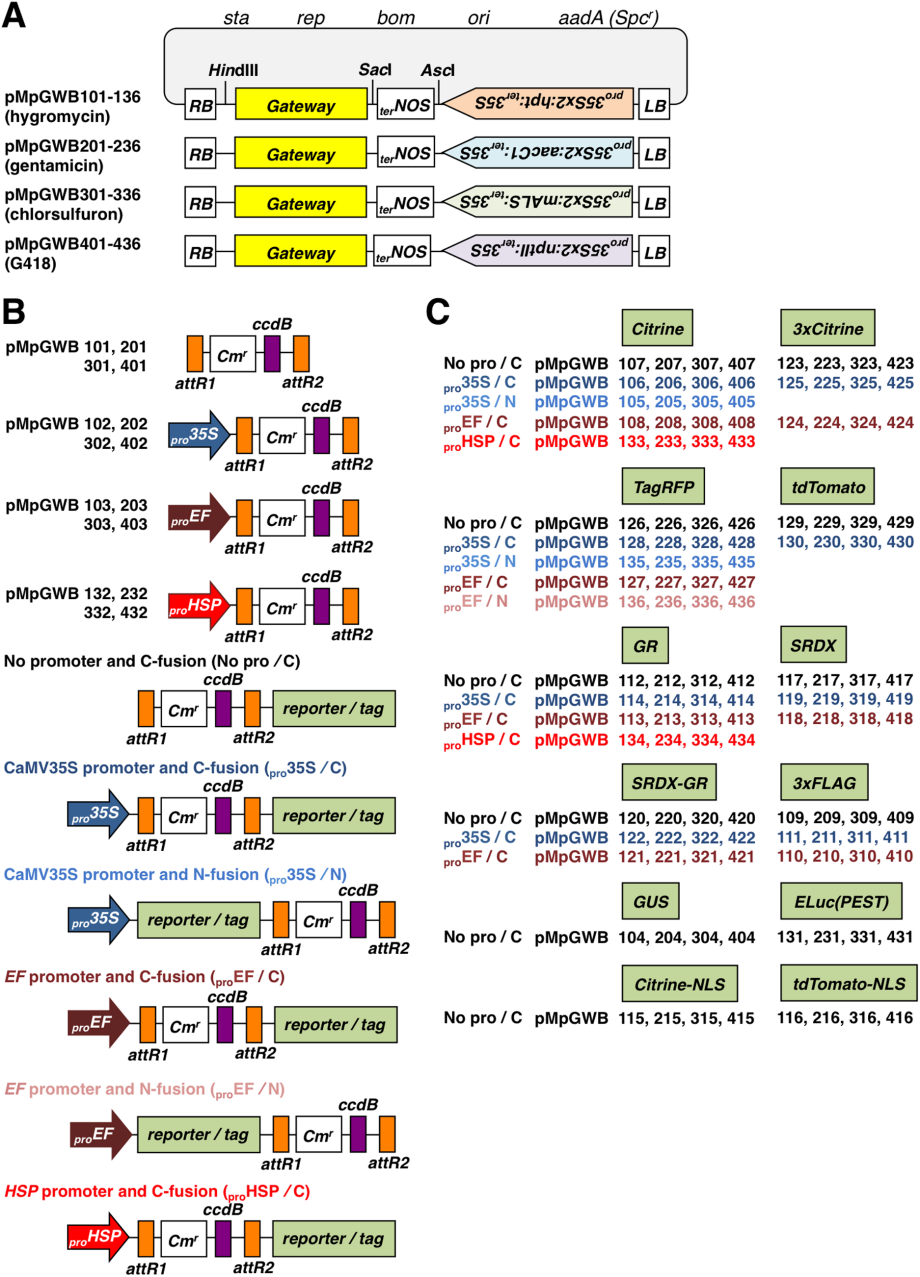
Schematic illustrations of pMpGWBs. A. Outline of pMpGWBs (pMpGWB101-136, pMpGWB201-236, pMpGWB301-336, and pMpGWB401-436 containing selection markers for Hyg, Gen, CS, and G418, respectively). Markers were placed in tail-to-tail orientation in relation to genes cloned by LR reaction. B. Structures of “*Gateway*” regions shown in (A). Only general structures are shown for vectors harboring fusion construct; *i*.*e*., no promoter and C-fusion (No pro/C), CaMV 35S promoter and C-fusion (_pro_35S/C), CaMV 35S promoter and N-fusion (_pro_35S/N), *EF* promoter and C-fusion (_pro_EF/C), *EF* promoter and N-fusion (_pro_EF/N), *MpHSP17*.*8A1* promoter and C-fusion (_pro_HSP/C). C. Reporters and tags used in pMpGWBs illustrated in (B). Vector number corresponds to combination of reporter/tag and fusion type. Citrine, synthetic yellow fluorescent protein; 3×Citrine, three repeats of Citrine fluorescent protein; TagRFP, tag red fluorescent protein; tdTomato; tandem dimer Tomato fluorescent protein; GR, ligand-binding domain of rat glucocorticoid receptor; SRDX, modified EAR motif plant-specific repression domain with strong repression activity; SRDX-GR, SRDX fused with GR; 3×FLAG, three repeats of FLAG tag; GUS, β-glucuronidase; ELuc(PEST), Emerald Luc (luciferase from click beetle) fused with PEST sequence; Citrine-NLS, Citrine fused with nuclear localization signal sequence; tdTomato-NLS, tdTomato fused with nuclear localization signal sequence. *RB*, right border; *LB*, left border; *sta*, region conferring stability in *Agrobacterium*; *rep*, broad host-range replication origin; *bom*, *cis*-acting element for conjugational transfer; *ori*, ColE1 replication origin. *Cm* ^*r*^, chloramphenicol-resistance marker (chloramphenicol acetyl transferase) used to select bacteria; *aadA*, spectinomycin-resistance marker (*Spc* ^*r*^) used to select bacteria; *ccdB*, negative selection marker.

In functional genetic studies, the construction of fusion genes is valuable for analyses of expression, protein localization, and protein–protein interactions *in vivo*. We have constructed Gateway binary vectors (pMpGWBs) for *M*. *polymorpha* based on the four binary vectors pMpGWB100, pMpGWB200, pMpGWB300, and pMpGWB400. The pMpGWB vectors allow genes to be fused to a variety of reporters and tags through a simple and uniform procedure using Gateway cloning technology [38]. The structure of the pMpGWBs is shown in Fig 3. The pMpGWB100 series carry the hygromycin-resistance marker (_*pro*_ *35S×2*:*hpt*:_*ter*_ *35S*). Similarly, the pMpGWB200, pMpGWB300, and pMpGWB400 series carry the gentamicin-resistance marker (_*pro*_ *35S×2*:*aacC1*:_*ter*_ *35S*), the CS-resistance marker (_*pro*_ *35S×2*:*mALS*:_*ter*_ *35S*), and the G418-resistance marker (_*pro*_ *35S×2*:*nptII*: _*ter*_ *35S*), respectively. All markers were placed in the tail-to-tail orientation in relation to the gene cloned by the LR reaction.

The last two digits of the plasmid names indicate the structures around the Gateway cassette that are common to the pMpGWBs. For example, pMpGWB101, pMpGWB201, pMpGWB301, and pMpGWB401 are simple cloning vectors with neither promoters nor reporters/tags. These vectors can be used in various experiments, for example, for complementation of mutations with genomic fragments.

pMpGWB102, pMpGWB202, pMpGWB302, and pMpGWB402 carry a standard CaMV 35S promoter (_*pro*_ *35S*) for constitutive expression in plants. pMpGWB103, pMpGWB203, pMpGWB303, and pMpGWB403 carry the endogenous *EF1α* promoter (_*pro*_ *EF*), which drives constitutive expression in *M*. *polymorpha* plants [34]. pMpGWB132, pMpGWB232, pMpGWB332, and pMpGWB432 carry the endogenous *MpHSP17*.*8A1* promoter (_*pro*_ *HSP*) for heat-inducible expression in *M*. *polymorpha* [35].

Fusion genes of promoter–GUS are usually used in histochemical and quantitative expression analyses, while promoter–luciferase fusions are useful for monitoring transcriptional responses in living cells. pMpGWB104, pMpGWB204, pMpGWB304, and pMpGWB404 are suitable for promoter analysis with the GUS reporter gene. pMpGWB131, pMpGWB231, pMpGWB331, and pMpGWB431 contain a luciferase gene from click beetle (*Photinus pyralis*) [39] fused to the mouse ornithine decarboxylase PEST sequence at the C-terminus. The *ELuc(PEST)* fusion gene was constructed to produce a promoter–luciferase assay with enhanced response dynamics [40]. pMpGWB115-116, pMpGWB215-216, pMpGWB315-316, and pMpGWB415-416 contain sequences encoding fluorescent proteins, Citrine or tdTomato, fused to a short amino acid sequence that contains the nuclear localization signal sequence (PKKKRKV) from the SV40 large T antigen [41]. These vectors were designed for monitoring promoter activity *in planta*. For promoter analysis, one would assume that the double-enhancer version of the CaMV 35S promoter, which drives marker genes in these pMpGWB vectors, might affect promoter activity in the same T-DNA. However, this does not appear to be the case in *M*. *polymorpha* as shown previously [13, 35].

Multiple fusion types are available for most reporters/tags. There are vectors for constitutive expression, driven by the CaMV 35S promoter, of proteins fused with reporters at the C-terminus (_pro_35S/C) or at the N-terminus (_pro_35S/N). There are vectors for expression of C-terminal fusion proteins driven by their own promoters (No pro/C). There are also vectors for the endogenous *EF1α* promoter-driven expression of proteins that are fused with a reporter at the C-terminus (_pro_EF/C) or at the N-terminus (_pro_EF/N), and those for the endogenous *MpHSP17*.*8A1* promoter-driven expression of proteins that are fused with a reporter at the C-terminus (_pro_HSP/C).

Fluorescent proteins and epitope tags are also useful for co-purification and immuno-detection experiments. Various kinds of fluorescent proteins with different spectral properties have been developed, and used to analyze the dynamics of protein localization *in planta*. The following vectors are suitable for expressing proteins in *planta* as protein-fusions with fluorescent reporters (Citrine, 3×Citrine, TagRFP, and tdTomato) [42–44]: pMpGWB105-108, pMpGWB123-130, pMpGWB133, pMpGWB135-136, pMpGWB205-208, pMpGWB223-230, pMpGWB233, pMpGWB235-236, pMpGWB305-308, pMpGWB323-330, pMpGWB333, pMpGWB335-336, pMpGWB405-408, pMpGWB423-430, pMpGWB433, and pMpGWB435-436. The vectors pMpGWB109-111, pMpGWB209-211, pMpGWB309-311, and pMpGWB409-411 are suitable for expressing proteins labeled with the 3×FLAG epitope tag at the C-terminus.

The vectors pMpGWB112-114, pMpGWB134, pMpGWB212-214, pMpGWB234, pMpGWB312-314, pMpGWB334, pMpGWB412-414, and pMpGWB434 were designed for expressing nuclear proteins, *i*.*e*. transcriptional regulators, as protein-fusions with the ligand-binding domain of the rat glucocorticoid receptor (GR). Using these constructs, the activity of transcription factors can be made conditional on the presence of steroid ligands, such as dexamethasone, as shown in previous studies [45–47].

The vectors pMpGWB117-119, pMpGWB217-219, pMpGWB317-319, and pMpGWB417-419 were designed for expressing transcriptional regulators tagged with the modified EAR motif plant-specific repression domain (SRDX; amino acid sequence LDLDLELRLGFA), which shows strong repression activity, at the C-terminus [48]. A chimeric repressor that is produced by fusing a transcription factor to SRDX suppresses the target genes of the transcription factor dominantly over the activity of endogenous and functionally redundant transcription factors [48]. pMpGWB120-122, pMpGWB220-222, pMpGWB320-322, and pMpGWB420-422 were designed for expressing transcriptional regulators tagged with both SRDX and GR at the C-terminus. Using these constructs, the activity of the chimeric transcriptional repressor can be made conditional on the presence of steroid ligands.

### Simultaneous transformation with multiple constructs

**Fig 4:**
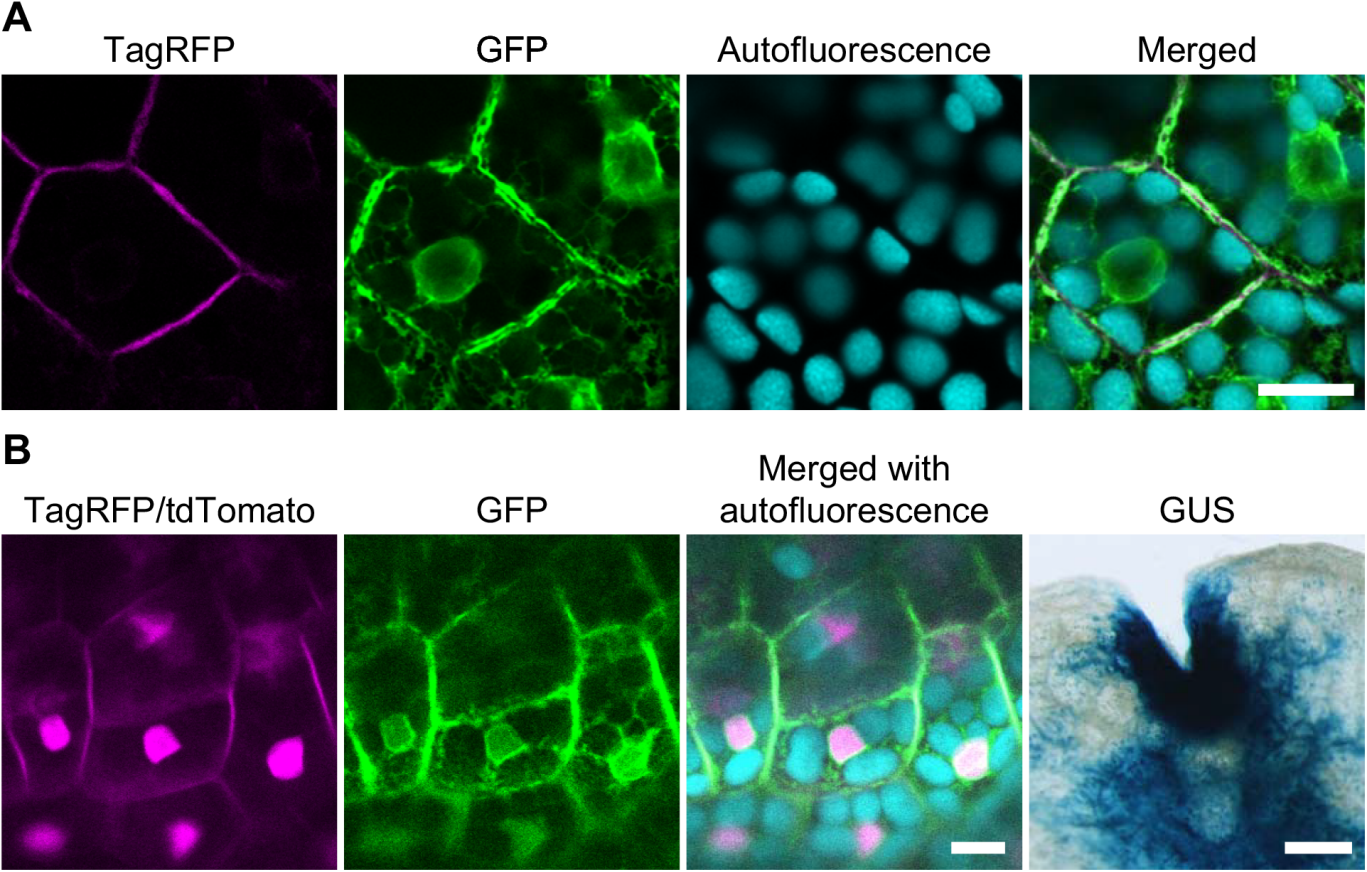
Simultaneous transformation with multiple pMpGWB vectors. A. Two binary vectors, pMpGWB103-TagRFP-LTI6b and pMpGWB303-SP-GFP-HDEL, were used simultaneously for *Agrobacterium*-mediated sporeling transformation with double selection. B. Four binary vectors, pMpGWB103-TagRFP-LTI6b, pMpGWB216-proEF, pMpGWB303-SP-GFP-HDEL, and pMpGWB404-proEF, were used simultaneously for transformation with quadruple selection. Separate and merged images for fluorescence from TagRFP or TagRFP/tdTomato, GFP, and chlorophyll autofluorescence of thallus epidermal cells and a micrograph for GUS staining in a representative line from each transformation are shown. Bars = 10 μm for fluorescence; 1 cm for GUS.

To test the performance of these vectors, we made two types of binary vectors: one designed to express a plasma membrane-localized fluorescent protein, TagRFP-LTI6b [49], under the control of _*pro*_ *EF* using pMpGWB103; and the other designed to express an endoplasmic reticulum-localized fluorescent protein, SP-GFP-HDEL [32], under the control of _*pro*_ *EF* using pMpGWB303. These binary vectors were separately introduced into *Agrobacterium* cells, and the respective transformant clones were used together to transform *M*. *polymorpha* sporelings. Sporelings co-cultured with the mixed *Agrobacterium* culture were subjected to hygromycin selection first, and then, to CS selection. Approximately 30% of transformants that passed the first hygromycin selection were resistant also to CS. Transgenic plants that survived selection with both hygromycin and CS were found to express the two proteins at their predicted subcellular locations (Fig 4A). We also examined triple and quadruple transformation using the two plasmids used above plus _*pro*_ *EF*-introduced pMpGWB216 (for expression of tdTomato-NLS) or _*pro*_ *EF*-introduced pMpGWB404 (for expression of GUS), or both. A portion (10–20%) of transformants that passed the first hygromycin selection showed resistance to either of the following combinatorial second selections: (i) hygromycin, gentamicin, and CS; (ii) hygromycin, CS, and G418; or (iii) all the four antibiotics/herbicide. As expected, expression of all transgenes in quadruple transformants was observed at predicted locations (Fig 4B).

Thus, the pMpGWB series are capable of driving proper expression. In addition, the four kinds of selection markers available in the pMpGWB system should provide options for transformation with multiple constructs simultaneously.

## Conclusion

We have developed a series of binary vectors for transformation of *M*. *polymorpha*. Four kinds of selection markers are now available for transgenesis of *M*. *polymorpha*. The pMpGWBs constructed in this study will be useful for efficient cloning in transgenic research, and will be particularly powerful for the construction of fusion genes and simultaneous introduction of multiple constructs. We have used various reporters and tags suitable for Marchantia research in these pMpGWBs.

In Arabidopsis, the Gateway vector series developed by Nakagawa et al. have been widely used and have accelerated the construction of transgenes [31,32,50]. It should be noted that the pMpGWBx00 binary vectors have unique *Hin*dIII/*Sac*I sites next to the nopaline synthase terminator sequence (_*ter*_ *NOS*). Consequently, the *Hin*dIII/*Sac*I fragments in the pGWB and R4pGWB series [31,32,50] that harbor Gateway cassettes and reporter/tag genes can be transferred into pMpGWBs by a single-step cloning procedure. Therefore, various Gateway cassettes and reporter/tags in the pGWB and R4GWB series (*i*.*e*., sGFP, G3GFP, mRFP, 6×His, 3×HA, 4×Myc, 10×Myc, GST, T7, and TAP) can be easily transferred into the pMpGWB series as needed. Similarly, the original Gateway cassette and reporters/tags in pMpGWBs (Citrine, 3×Citrine, Citrine-NLS, tdTomato, tdTomato-NLS, GR, SRDX, SRDX-GR, ELuc(PEST), and 3×FLAG) can be readily transferred into pGWBs. Although the pMpGWBs were developed for transgenesis experiments in *M*. *polymorpha*, they could also be used for other plants in which the _*pro*_ *35S×2*-driven selection markers are effective, such as monocots.

## Supporting Information

S1 Fig Dose-dependent effect of antibiotics/herbicide on growth of *M*. *polymorpha*. Wild-type (Takaragaike-1) gemmae were plated on medium containing 0–200 mg/l gentamicin (Gen; A), 0–1 μM chlorsulfuron (CS; B), or 0–10 mg/l G418 (C), and incubated for 10 d at 22°C under continuous light.
(PDF)

S2 Fig Schematic illustration of pMpGWB vector backbones. Restriction enzyme sites available for linearization of respective plasmids are indicated. *RB*, right border; *LB*, left border; *sta*, region conferring stability in *Agrobacterium*; *rep*, broad host-range replication origin; *bom*, *cis*-acting element for conjugational transfer; *ori*, ColE1 replication origin. *aadA*, spectinomycin-resistance marker (*Spc* ^*r*^) used for bacterial selection.
(PDF)

S1 Table Primers used in this study. (XLSX)

S1 Text pMpGWBs vector construction. (DOCX)

## Abbreviations

aacC1: gentamicin 3'-acetyltransferase
CaMV: cauliflower mosaic virus
CS: chlorsulfuron
ELuc(PEST): Emerald Luc (luciferase from click beetle) fused with PEST sequence
GR: glucocorticoid receptor domain
GUS: β-glucuronidase
hpt: hygromycin phosphotransferase
HSP: heat-shock protein
LB: left border
mALS: mutated acetolactate synthase gene
NLS: nuclear localization signal
nptII: neomycin phosphotransferase II
_pro_35S: CaMV 35S promoter
_pro_35S×2: double enhancer version of CaMV 35S promoter
_pro_EF: promoter of *ELONGATION FACTOR1α*
_pro_HSP: promoter of *MpHSP17*.*8A1*
RB: right border
SRDX: modified EAR motif plant-specific repression domain showing strong repression activity
TagRFP: tag red fluorescent protein
tdTomato: tandem dimer Tomato
_ter_35S: 3′UTR of CaMV 35S gene
_ter_NOS: terminator sequence of nopaline synthase gene
