# Supplementary material for: Development of Gateway Binary Vector Series with Four Different Selection Markers for the Liverwort Marchantia polymorpha
Source: PLoS One. 2015 Sep 25;10(9):e0138876. doi: 10.1371/journal.pone.0138876 (PMC4583185; doi:10.1371/journal.pone.0138876)
Supplement: S1 Fig — Wild-type (Takaragaike-1) gemmae were plated on medium containing 0–200 mg/l gentamicin (Gen; A), 0–1 μM chlorsulfuron (CS; B), or 0–10 mg/l G418 (C), and incubated for 10 d at 22°C under continuous light. (PDF) [file pone.0138876.s001.pdf]

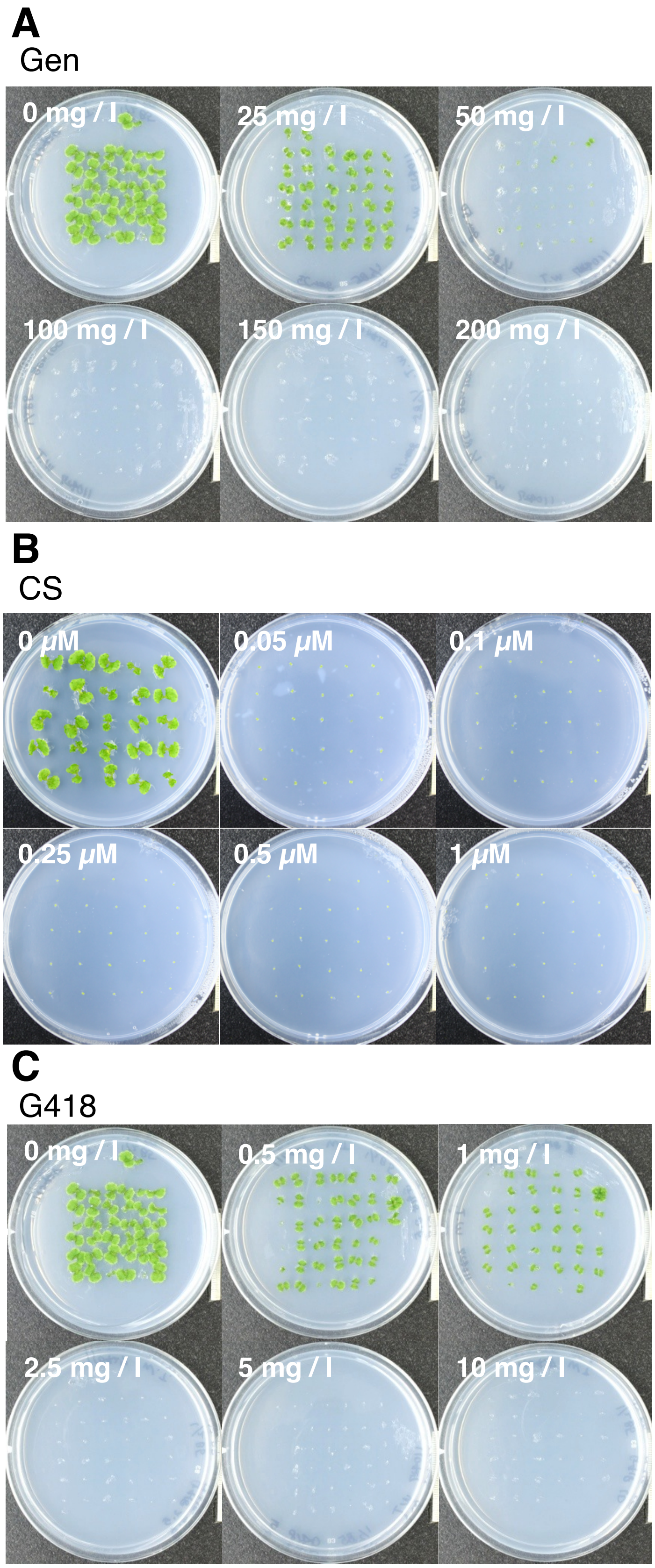

S1 Fig. Dose-dependent effect of antibiotics/herbicides on growth of *M. polymorpha*.
Wild-type (Takaragaike-1) gemmae were plated on medium containing 0–200 mg/l gentamicin (Gen; A), 0–1 μM chlorsulfuron (CS; B), or 0–10 mg/l G418 (C), and incubated for 10 d at 22°C under continuous light.
