# Supplementary material for: Development of Gateway Binary Vector Series with Four Different Selection Markers for the Liverwort Marchantia polymorpha
Source: PLoS One. 2015 Sep 25;10(9):e0138876. doi: 10.1371/journal.pone.0138876 (PMC4583185; doi:10.1371/journal.pone.0138876)
Supplement: S2 Fig — Restriction enzyme sites available for linearization of respective plasmids are indicated. RB, right border; LB, left border; sta, region conferring stability in Agrobacterium; rep, broad host-range replication origin; bom, cis-acting element for conjugational transfer; ori, ColE1 replication origin. aadA, spectinomycin-resistance marker (Spc r) used for bacterial selection. (PDF) [file pone.0138876.s002.pdf]

pMpGWB100

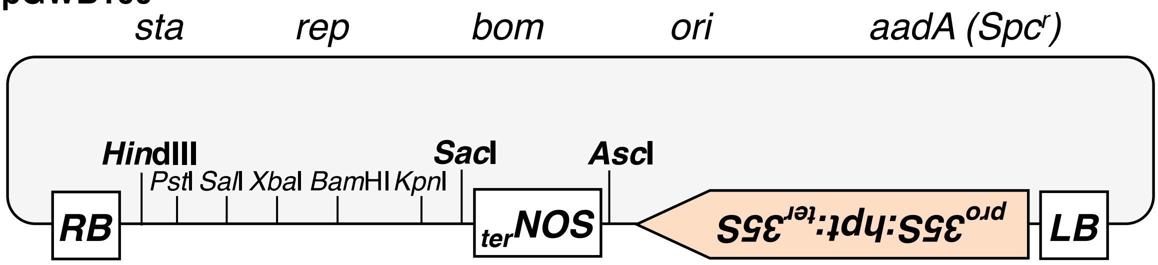

Selection for bacteria: Spectinomycin (100 mg / L)
Selection for plant: Hygromycin (10 mg / L)

pMpGWB200

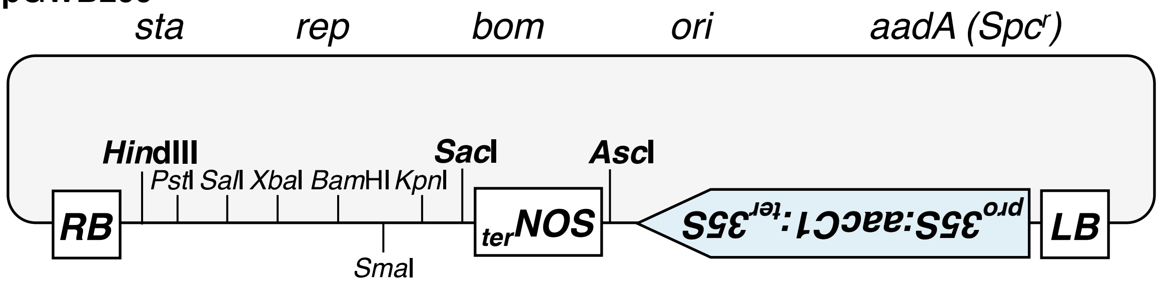

Selection for bacteria: Spectinomycin (100 mg / L)
Selection for plant: Gentamycin (100 mg / L)

pMpGWB300

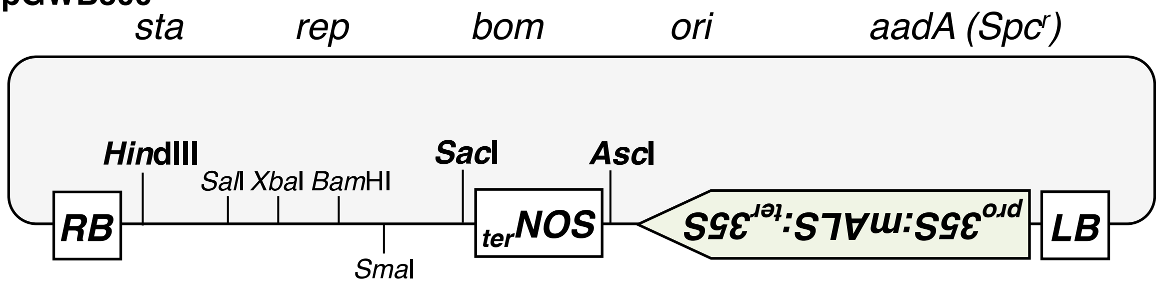

Selection for bacteria: Spectinomycin (100 mg / L)
Selection for plant: Chlorsulfuron (0.5 μM)

pMpGWB400

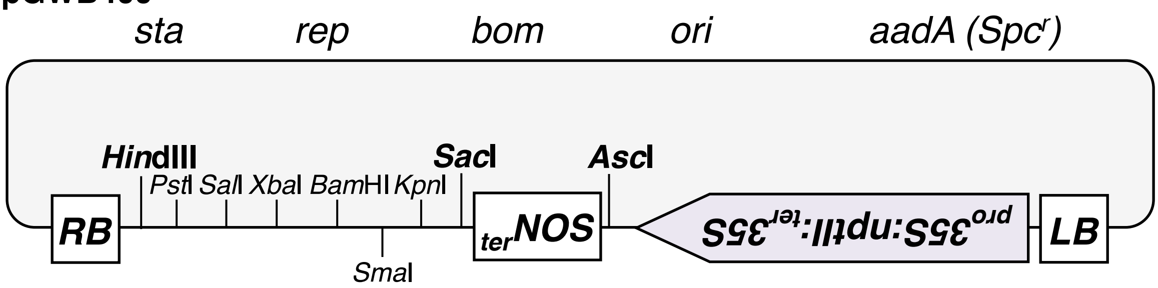

Selection for bacteria: Spectinomycin (100 mg / L)
Selection for plant: G418 (5 mg / L)

S2 Fig. Schematic illustration of pMpGWB vector backbones.
Restriction enzyme sites available for linearization of respective plasmids are indicated. *RB*, right border; *LB*, left border; *sta*, region conferring stability in *Agrobacterium*; *rep*, broad host-range replication origin; *bom*, *cis*-acting element for conjugational transfer; *ori*, ColE1 replication origin. *aadA*, spectinomycin-resistance marker (*Spc^r*) used for bacterial selection.
