# Supplementary material for: Development of Gateway Binary Vector Series with Four Different Selection Markers for the Liverwort Marchantia polymorpha
Source: PLoS One. 2015 Sep 25;10(9):e0138876. doi: 10.1371/journal.pone.0138876 (PMC4583185; doi:10.1371/journal.pone.0138876)
Supplement: S1 Text — (DOCX) [file pone.0138876.s004.docx]

**S1 Text**

**pMpGWBs vector construction**

To construct the pMpGWBx01 series (x = 1, 2, 3, or 4), the *Xba*I–*Sac*I fragment of pGWB1 [1] was transferred into the same sites of the corresponding pMpGWBx00 vectors.

pUGW0 and pUGW2 [1] were used to construct a series of intermediate plasmids. To construct the no-promoter version of an intermediate plasmid, the CaMV 35S promoter was removed by digesting pUGW2 with *Xba*I, followed by self-ligation, to generate pUGW2Δ35S. A region (−1,735 to −6, where A in the putative initiation codon is +1) of the upstream regulatory sequence of the endogenous *ELONGATION FACTOR1α* gene [2] was amplified by PCR using *M. polymorpha* genomic DNA as the template with the primer set MpEF_L_HindIII and MpEF_R_SpeI. This region of the upstream regulatory sequence contains one intron. The amplified PCR fragment was cloned into the *Hin*dIII–*Xba*I site of pUGW0 and pUGW2 to generate pKIGW3 and pKIGW4, respectively. The *Hin*dIII–*Sac*I fragment of pUGW2 [1] and pKIGW4 was transferred into the same sites of the corresponding pMpGWBx00 vectors to generate the pMpGWBx02 and pMpGWBx03 series, respectively.

The pMpGWBx04 series containing the GUS reporter were constructed by transferring the *Hin*dIII–*Sac*I fragment of pGWB3 [1] into the same sites of the corresponding pMpGWBx00 vectors.

The coding sequence of Citrine [3] (kindly provided by Roger Y. Tsien, University of California at San Diego) was amplified by PCR with the primer set Citrine_L and Citrine_Rns, and cloned into the *Aor*51HI site of pUGW0 to construct pKIGW5. Similarly, the coding sequence of Citrine was amplified by PCR with the primer set Citrine_L and Citrine_Rs and cloned into the *Aor*51HI site of pUGW2 and pUGW2Δ35S to construct pKIGW6 and pKIGW7, respectively. Finally, the coding sequence of Citrine with a GGSGGS linker sequence at the N-terminus was amplified by PCR with pUGW2-GGS2-FP_IF_F and pUGW2-FP_IF_R and cloned into the *Aor*51HI site of pKIGW4 using an In-Fusion HD cloning kit (Clontech) to construct pKRN38. The *Hin*dIII–*Sac*I fragment of pKIGW5, pKIGW6, and pKIGW7 was transferred into the same sites of the corresponding pMpGWBx00 vectors to generate the pMpGWBx05, pMpGWBx06, and pMpGWBx07 series, respectively. The *Hin*dIII–*Hin*dIII fragment (second *Hin*dIII site was accidentally generated in the primer pUGW2-FP_IF_R) of pKRN38 was transferred into the *Hin*dIII site of the corresponding pMpGWBx00 vectors to generate the pMpGWBx08 series.

The coding sequence of 3×FLAG (DYKDHDGDYKDHDIDYKDDDDK) was amplified by PCR with the phosphorylated primer set 3×FLAG_F and 3FLAG_SacI_R, digested with *Sac*I, and then cloned into the *Aor*51HI–*Sac*I sites of pUGW2Δ35S, pKIGW4, and pUGW2. The *Hin*dIII–*Sac*I fragment of the resulting plasmids was transferred into the same sites of the pMpGWBx00 vectors to generate the pMpGWBx09, pMpGWBx10, and pMpGWBx11 series, respectively.

The GR coding sequence was amplified by PCR using a plasmid containing LhGR fragment [4] as the template with the phosphorylated primer set GR_L and GR_SacI_R2, digested with *Sac*I, and then cloned into the *Aor*51HI–*Sac*I sites of pUGW2Δ35S, pKIGW4, and pUGW2. Two *Hin*dIII sites within the GR sequence in the resulting plasmids were destroyed with synonymous substitutions using PCR-based site-directed mutagenesis sequentially with two primer sets; GR_mHind_F and GR_mHind_R, and GR_mHind_F2 and GR_mHind_R3. The *Hin*dIII–*Sac*I fragment of the modified plasmids was transferred into the same sites of the pMpGWBx00 vectors to generate the pMpGWBx12, pMpGWBx13, and pMpGWBx14 series, respectively.

The coding sequences of Citrine with the SV40 NLS sequence at the C-terminus (Citrine-NLS) and its tdTomato [5] version (tdTomato-NLS) were both amplified by PCR with the phosphorylated primer set YFP-ATG-F-070724 and tdTomato_NLS_R, digested with *Sac*I, and then cloned into the *Aor*51HI–*Sac*I sites of pUGW2Δ35S. The *Hin*dIII–*Sac*I fragment of the resulting plasmids was transferred into the same sites of the pMpGWBx00 vectors to generate the pMpGWBx15 and pMpGWBx16 series, respectively.

The coding sequence of GR with the SRDX sequence [6] at the N-terminus (SRDX-GR) was amplified by PCR using pMpGWB112 as the template with the phosphorylated primer set SRDX_GR_F and GR_SacI_R2, digested with *Sac*I, and then cloned into the *Aor*51HI–*Sac*I sites of pUGW2Δ35S, pKIGW4, and pUGW2. The *Hin*dIII–*Sac*I fragment of the resulting plasmids was transferred into the same sites of the pMpGWBx00 vectors to generate the pMpGWBx20, pMpGWBx21, and pMpGWBx22 series, respectively.

The SRDX sequence was then amplified by PCR using pMpGWB120 as the template with primer set SRDX_IF_F and SRDX_IF_R and cloned into the *Aor*51HI site of pUGW2Δ35S, pKIGW4, and pUGW2 using an In-Fusion HD Cloning Kit. The *Hin*dIII–*Sac*I fragment of the resulting plasmids was transferred into the same sites of the pMpGWBx00 vectors to generate the pMpGWBx17, pMpGWBx18, and pMpGWBx19 series, respectively.

The pMpGWBx23, pMpGWBx24, and pMpGWBx25 series containing 3×Citrine were constructed as follows. The Citrine coding sequence was amplified by PCR with the primer set pUGW2-GGS2-FP_IF_F and pUGW2-FP_tandem_IF_R and cloned into the *Aor*51HI site of pUGW2Δ35S and pUGW26 using an In-Fusion HD Cloning Kit. The *Aor*51HI site (regenerated in the primer) of the resulting plasmids as well as pKRN38 was used for In-Fusion cloning of the same *Citrine* PCR fragment. The resulting plasmids were used for one more round of In-Fusion cloning of the same *Citrine* PCR fragment at the regenerated *Aor*51HI site. The *Hin*dIII–*Hin*dIII fragment of the derivatives containing the 3×Citrine sequence was transferred into the *Hin*dIII site of the pMpGWBx00 vectors to generate the pMpGWBx23, pMpGWBx25, and pMpGWBx24 series, respectively.

The coding sequence of TagRFP [7] was amplified by PCR with the primer set pUGW2-GGS2-TagRFP_IF_F and pUGW2-TagRFP_IF_R and cloned into the *Aor*51HI site of pUGW2Δ35S and pUGW2 using an In-Fusion HD Cloning Kit. The *Hin*dIII–*Sac*I fragment of the resulting plasmids was transferred into the same sites of the pMpGWBx00 vectors to generate the pMpGWBx26 and pMpGWBx28 series, respectively. The *Pme*I–*Sal*I fragment containing the *EF1α* promoter and the Gateway cassette of pMpGWB103 was transferred into the same sites of pMpGWBx26 to generate the pMpGWBx27 series.

The coding sequence of tdTomato was amplified by PCR with the primer set pUGW2-GGS2-FP_IF_F and pUGW2-FP_IF_R and cloned into the *Aor*51HI site of pUGW2Δ35S and pUGW2 using an In-Fusion HD Cloning Kit. The *Hin*dIII–*Hin*dIII fragment of the resulting plasmids was transferred into the *Hin*dIII site of the pMpGWBx00 vectors to generate the pMpGWBx29 and pMpGWBx30 series, respectively.

The coding sequence of *Eluc(PEST)* was amplified by PCR using pELuc(PEST)-test (Toyobo) as the template with the primer set pUGW2-ELuc_IF_F and pUGW2-ELuc_IF_R and cloned into the *Aor*51HI site of pMpGWB301 to generate pMpGWB331. The *Hin*dIII–*Sac*I fragment of pMpGWB331 was transferred into the same sites of pMpGWB101, pMpGWB201, and pMpGWB401 to generate pMpGWB131, pMpGWB231, and pMpGWB431, respectively.

The *MpHSP17.8A1* promoter was amplified by PCR using pENTRD-MpHSP17.8A1pro (Nishihama et al., 2015 under revision) as the template with the primer set pUGW2-MpHSP17.8_IF_F and pUGW2-MpHSP17.8_IF_R. The PCR product was cloned into the *Xba*I sites of pMpGWBx01, pMpGWBx06, and pMpGWBx12 to generate the pMpGWBx32 series (no tag), the pMpGWBx33 series (C-terminal Citrine fusion), and the pMpGWBx34 series (C-terminal GR fusion), respectively.

For vectors to produce an N-terminal TagRFP fusion, the coding sequence of TagRFP was amplified by PCR with the primer set TagRFP_ATG_pMpx02_IF_F and TagRFP_noSTP_pMpx02_IF_R and cloned into the *Hin*dIII–*Xba*I site of pMpGWBx02 vectors. The *Hin*dIII site of the resulting plasmid was used to clone promoters: the CaMV 35S promoter that was PCR-amplified using the primer set 35S.pBI121_IF_pMp_Hind_F and 35S.pBI121_IF_pMpRFP_Hind_R; and the *EF1α* promoter that was PCR-amplified with the primer set EFpro_IF_pMp_Hind_F and EFpro_IF_pMpRFP_Hind_R. The addition of the CaMV 35S promoter and the *EF1α* promoter generated the pMpGWBx35 and pMpGWBx36 series, respectively.

**References for S1 Text**

1. Nakagawa T, Kurose T, Hino T, Tanaka K, Kawamukai M, Niwa Y, et al. Development of series of gateway binary vectors, pGWBs, for realizing efficient construction of fusion genes for plant transformation. J Biosci Bioeng. 2007; 104: 34-41.doi: 10.1263/jbb.104.34 PMID: 17697981.

2. Althoff F, Kopischke S, Zobell O, Ide K, Ishizaki K, Kohchi T, et al. Comparison of the MpEF1α and CaMV35 promoters for application in Marchantia polymorpha overexpression studies. Transgenic Res. 2014; 23: 235-244.doi: 10.1007/s11248-013-9746-z PMID: 24036909.

3. Shaner NC, Steinbach PA, Tsien RY. A guide to choosing fluorescent proteins. Nat Methods. 2005; 2: 905-909. doi:10.1038/nmeth819 PMID: 16299475.

4. Craft J, Samalova M, Baroux C, Townley H, Martinez A, Jepson I, Tsiantis M, Moore I. New pOp/LhG4 vectors for stringent glucocorticoid-dependent transgene expression in Arabidopsis. Plant J. 2005; 41: 899-918. doi: 10.1111/j.1365-313X.2005.02342.x. PMID: 15743453.

5. Day RN, Davidson MW. The fluorescent protein palette: tools for cellular imaging. Chem Soc Rev. 2009; 38: 2887-2921. doi: 10.1039/b901966a PMID: 19771335.

6. Mitsuda N, Matsui K, Ikeda M, Nakata M, Oshima Y, Nagatoshi Y, et al. CRES-T, an effective gene silencing system utilizing chimeric repressors. Methods Mol Biol. 2011; 754: 87-105. doi: 10.1007/978-1-61779-154-3_5 PMID: 21720948.

7. Merzlyak EM, Goedhart J, Shcherbo D, Bulina ME, Shcheglov AS, Fradkov AF, et al. Bright monomeric red fluorescent protein with an extended fluorescence lifetime. Nat Methods. 2007; 4: 555-557. doi: 10.1038/nmeth1062 PMID: 17572680.
